# Phenotypic correction of Fanconi anemia cells in the murine bone marrow after carrier cell mediated delivery of lentiviral vector

**DOI:** 10.1186/s13287-016-0431-z

**Published:** 2016-11-19

**Authors:** Santhosh Chakkaramakkil Verghese, Natalya A. Goloviznina, Peter Kurre

**Affiliations:** 1 Department of Pediatrics, Oregon Health & Science University, Portland, OR 97239 USA; 2 Papé Family Pediatric Research Institute, Oregon Health & Science University, L321, Portland, OR 97239 USA; 3 Present address: Molecular, Cellular, Developmental Biology and Genetics, University of Minnesota, Minneapolis, MN 55455 USA

**Keywords:** Fanconi Anemia, Gene Therapy, In situ gene delivery, Lentiviral vector, Hematopoietic stem cells, FA gene therapy

## Abstract

Fanconi anemia (FA) is an autosomal-recessive disorder associated with hematopoietic failure and it is a candidate for hematopoietic stem cell (HSC)-directed gene therapy. However, the characteristically reduced HSC numbers found in FA patients, their ineffective mobilization from the marrow, and re-oxygenation damage during ex vivo manipulation have precluded clinical success using conventional in vitro approaches. We previously demonstrated that lentiviral vector (LV) particles reversibly attach to the cell surface where they gain protection from serum complement neutralization. We reasoned that cellular delivery of LV to the bone marrow niche could avoid detrimental losses during FA HSC mobilization and in vitro modification. Here, we demonstrate that a VSV-G pseudotyped lentivector, carrying the FANCC transgene, can be transmitted from carrier to bystander cells. In cell culture and transplantation models of FA, we further demonstrate that LV carrier cells migrate along SDF-1α gradients and transfer vector particles that stably integrate and phenotypically correct the characteristic DNA alkylator sensitivity in murine and human FA-deficient target bystander cells. Altogether, we demonstrate that cellular homing mechanisms can be harnessed for the functional phenotype correction in murine FA hematopoietic cells.

**Electronic supplementary material:**

The online version of this article (doi:10.1186/s13287-016-0431-z) contains supplementary material, which is available to authorized users.

## Introduction

Fanconi anemia (FA) is a multisystem disorder resulting from mutations in one of at least 22 genes that encode the FANC family of DNA repair proteins [1, 2]. For most patients, the associated bone marrow failure presents the most pressing clinical issue. The hematopoietic stem cell (HSC) attrition that underlies the typical cytopenias appears to reflect a combination of accrued DNA damage and an exaggerated susceptibility to innate immunity or aldehyde detoxification [3, 4]. Curative allogeneic stem cell transplantation, however, is available only to patients with suitably matched related or unrelated donors, and places them at risk of procedure-related complications [5, 6]. As an alternative, several groups are working to develop gene therapy methods to stably correct the patient’s own HSCs, using retro- or lentiviral vectors (LVs) [7, 8]. HSC-directed gene therapy has been successfully used to treat several hematologic and immunodeficiency disorders [9], but FA patients present unique challenges. Typically, the bone marrow (BM) cellularity of the patient is reduced, HSC numbers following G-CSF mobilization are low, and repopulation properties are further lost during ex vivo transduction culture [10–12]. Direct injection of viral vector particles into the BM has been proposed to bypass ex vivo manipulation of FA HSCs, but this strategy leads to rapid dilutional losses, complement neutralization, and vector sensitization [13, 14].

Several classes of enveloped viruses, including human T-cell leukemia virus (HTLV-1), form viral ‘biofilms’ that non-specifically attach virion particles to cells [14]. Indeed, direct biofilm-assisted cell-cell transfer increases viral transmission and systemic distribution to distant tissue sites [15, 16]. Cellular capture of human immunodeficiency virus (HIV) particles was also observed as a mode of HIV transmission across genital and gastrointestinal mucosal surfaces [17]. We previously reported that cell surface-bound HIV-1-derived LV particles gain protection from serum neutralization and transfer between cells with relatively greater efficiency compared with several cell-free virions [18, 19]. Here, we exploited those observations in a strategy that combines in vitro exposure of LV to “carrier” cells that traffic to the bone marrow, thereby minimizing in vitro manipulation of FA HSCs while achieving stable transduction through cellular in situ delivery. To simulate the key steps in this process, we systematically analyzed the role of chemotaxis to promote carrier cell migration to the hematopoietic niche. Given its critical role in BM homing, C-X-C chemokine receptor type 4 (CXCR4)-expressing leukemic cells L1210 and murine progenitors were used as carrier cells to demonstrate chemotactic migration in vitro and in vivo, respectively. Radiation of carrier cells prior to vector binding was used to avoid the persistence of carrier cells after vector delivery to recipient cells. Results showed that carrier cells can migrate along a chemotactic gradient of stromal derived factor-1α (SDF-1α) and traffic *FANCC* expressing LV to *Fancc* ^*–/–*^ murine hematopoietic stem and progenitor cell (HSPC) target cells, with subsequent transduction (TD) and expansion under selection pressure.

## Results

### In vitro cell-cell transfer of lentiviral vector

**Fig. 1:**
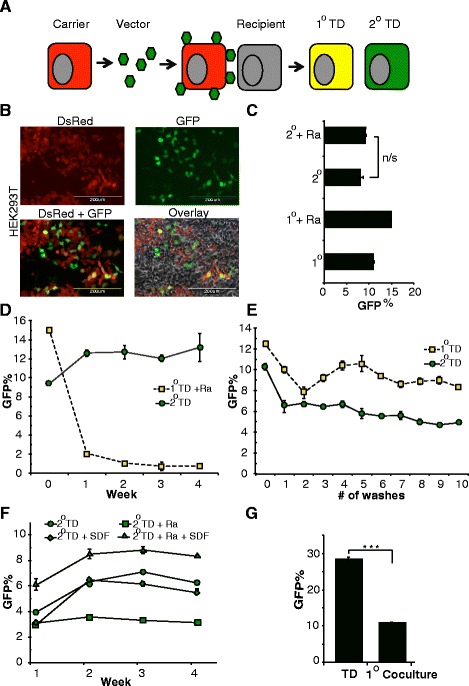
Factors affecting 2° TD. **a** Schematic representation of experimental design. DsRed expressing 293 T cells were used as carrier cells incubated with LV-GFP for 3 h followed by washes. The vector-coated carrier cells are then incubated overnight with 293 T cells in 1:1 ratio. Primary transduced (*1° TD*) cells will be double-positive for GFP and DsRed and secondary transduced (*2° TD*) cells will be GFP-positive. **b** Microscopic imaging of 1° TD and 2° TD HEK293T cells at 48 h post-transduction. **c** FACS analysis for fluorescent protein expression in HEK293T cell cultures to observe the fate of transduced cells (*n* = 3). **d** Long-term analysis of 2° TD and the fate of irradiated carrier cell (shown as *Ra*) measured by FACS. **e** Rate of 2° TD is measured after several washes of 1° TD cells with phosphate-buffered saline (PBS). **f** 2° TD in 293 T cells after transwell migration of murine L1210 cells towards the SDF-1α gradient. L1210-DsRed carrier cells were irradiated and incubated with LV-GFP for 3 h followed by washes. The vector-coated carrier cells are then incubated overnight with 293 T cells in 1:1 ratio. Cells were analyzed for up to 4 weeks. **g** FACS analysis of primary transduction rate in carrier cells (*n* = 3; ***p > 0.0001). *GFP* green fluorescent protein, *n/s* not significant, *SDF* stromal-derived factor

A lentiviral vector (LVCG) expressing GFP was used to measure the cell-cell transfer rate of vector particles in vitro. Carrier cells were generated by transducing human embryonic kidney cell line (HEK293T) with a DsRed expressing lentiviral vector (LV-DsRed) and enriched to purity by flow cytometric sorting. Primary transduction (1° TD) and secondary transduction (2° TD) to the bystander cells are detected based on the reporter protein expression in the transduced cells (Fig. 1a). In this experimental set-up, four fluorescence protein expression patterns could be observed: non-transduced carrier 293 T-DsRed cells, non-transduced wild-type 293 T cells, primary transduced (1° TD) 293 T (DsRed + GFP) cells, and secondary transduced (2° TD) 293 T-GFP cells (Fig. 1b). Radiation was used to selectively eliminate the carrier cells after 2° TD. Results show that the irradiation (Ra) of carrier cells had no significant impact on vector transfer to 2° recipient cells (Fig. 1c). Cells were maintained in culture for up to 4 weeks to analyze both 1° and 2° transduced cells. The projected depletion of irradiated carrier cells over time and the stability of transgene expression from integrated lentiviral vector was further confirmed by analyzing long-term culture (Fig. 1d).

To assess the stability of vector attachment to carrier cells, cells incubated with vector were washed repeatedly, and followed each time by co-culture with the recipient cells. The number of washes did not appear to significantly affect the rate of secondary transduction, suggesting that LV biofilms are not easily disrupted during manipulation prior to contact with recipient cells (Fig. 1e). To simulate 2° TD events after migration, we used a murine leukemia cell line, L1210, which constitutively overexpresses the chemokine receptor CXCR4. Cells with CXCR4 receptor expression exhibit chemotaxis towards the SDF-1α. 293 T cells in SDF-1α supplemented medium were plated in the bottom chamber of the transwell plate to facilitate 2° transduction after migration. Results indicate successful migration of L1210 cells along an SDF-1α gradient to the recipient 293 T cells (Fig. 1f). Given the direct competition between carrier and recipient cells for uptake and transduction by vector particles, we observed anticipated losses to 1° TD on carrier cells that occur during the course of cell-to-cell transfer of vector particles for 2° TD recipient cells (Fig. 1g). Overall, the experimental model of 2° TD after migration of irradiated carrier cells supports its potential for in situ gene delivery of therapeutic transgenes.

### Functional correction in *Fancc* defective cells in vitro

**Fig. 2:**
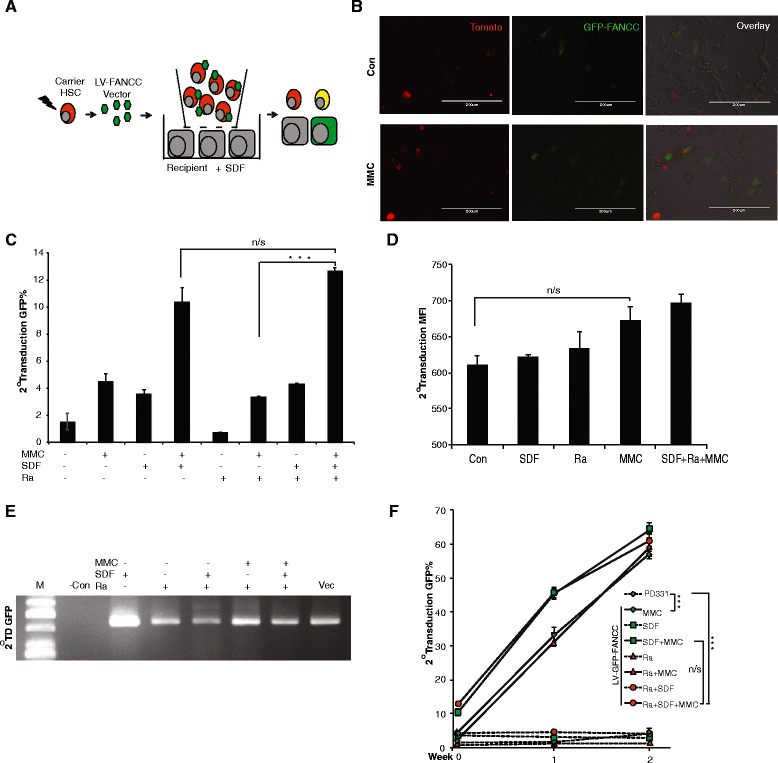
In vitro genetic correction and expansion of 2° TD *FANCC* ^*–/–*^ cells. **a** Diagram depicting the experimental steps of 2° TD in vitro. Tomato-positive HSPC cells were used as carrier cells harboring the LV-GFP-FANCC vector to migrate across the transwell membrane to the bottom chamber for 2° TD on the PD331 cells. **b** Microscopic imaging of 2° TD by using Tomato HSPC as a carrier and FANCC^*–/–*^ PD331 as recipient cells. **c** Measurement of 2° TD with experimental variables including radiation, DNA damage by MMC, and chemotaxis by SDF-1α. The combination of SDF-1α, radiation, and MMC were also used as different parameters that affect 2° TD directly or indirectly (*n* = 6; ***p > 0.0001). **d** Mean florescent intensity (*MFI*) as a measure of transgene expression. **e** PCR analysis of LV-GFP-FANCC status. **f** Expansion of FANCC-corrected 2° TD PD331 cells under MMC selection pressure (*n* = 3). *Con* control, *GFP* green fluorescent protein, *HSC* hematopoietic stem cell, *MMC* mitomycin-C, *n/s* not significant, *Ra* irradiation, *SDF* stromal-derived factor

Bystander cell transduction by LV particles using carrier cell delivery has the potential for therapeutic phenotypic correction of FA target cells located in an internal tissue compartment. Here, we modeled cellular delivery by using vector-bound HSPCs as carrier cells migrating by chemotaxis towards PD331, a human *FANCC* ^*–/–*^ fibroblast recipient cell line maintained in SDF-1α containing medium (Fig. 2a). Primary progenitor cells were used from Tomato protein-expressing transgenic animals [20] as carrier cells along with an HIV-based lentiviral vector LV-GFP-FANCC that expresses a GFP reporter and human *FANCC* for the phenotypic rescue. Co-culture of HSPC-Tomato cells carrying vector with PD331 cells resulted in the 2° TD of PD331 cells, indicated by GFP-FANCC-positive PD331 cells (Fig. 2b).

Case reports suggest that selective clonal expansion of spontaneously corrected HSPCs in FA patients can correct peripheral blood cytopenias [21, 22]. To mimic such a selection, we cultured phenotypically corrected FA cells in the presence of the DNA alkylating agent mitomycin-C (MMC). Results demonstrate significant 2° TD in the presence of SDF-1α while MMC enriches the transduced cells post-delivery (Fig. 2c). The FA pathway is known to be activated in response to MMC-induced DNA damage [21]. A gain in mean florescent intensity (MFI) of GFP from the GFP-FANCC fusion protein cassette was observed (Fig. 2d) in 2° transduced PD331 cells, suggesting phenotypic rescue by the GFP-FANCC transgene expression. Genomic DNA was extracted from the 2° transduced PD331 cells to analyze the vector DNA status in these cells by PCR using primer combination of poly purine tract (PPT Forward primer) from vector backbone and transgene (GFP-Reverse primer) expression unit. Proviral integration and the status of GFP-FANCC transgene cassette was confirmed in these 2° TD PD331 cells (Fig. 2e). When the 2° transduced PD331 cells are maintained in the presence of MMC, the ongoing selection pressure enriched the PD331-GFP-FANCC cells rapidly (Fig. 2f). The successful selection of corrected FA phenotype cells in vitro suggests the feasibility of translating this approach to an in vivo murine model of FA.

### In vivo gene correction of Fancc-deficient hematopoietic cells via cellular delivery of LV

**Fig. 3:**
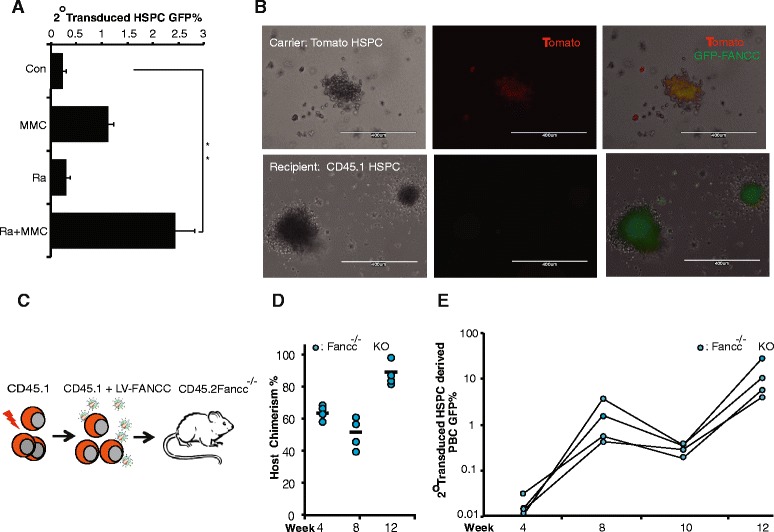
In situ gene delivery in *FANCC* ^*–/–*^ murine model. **a** Measurement of 2° TD by FACS between mT/mG HSPC as carrier cells and CD45.1 HSPCs as recipient cells (*n* = 3, **p < 0.001) **b** 1° and 2° transduced colony forming units (CFUs) expressing the corresponding florescent proteins. **c** Schematic diagram of in situ gene delivery model for FANCC correction and expansion of corrected cells. **d** Chimerism of the recipient FANCC^–/–^ CD45.2 cells up to 3 months post-transplant (*n* = 4) **e** GFP-FANCC expression in the recipient (CD45.2) cells up to 3 months post-transplant indicating 2° TD events. Irradiation to induce selection pressure by DNA repair was given at weeks 6 and 11. *Con* control, *GFP* green fluorescent protein, *HSPC* hematopoietic stem and progenitor cell, *KO* knockout, *MMC* mitomycin-C, *PBC* peripheral blood cells, *Ra* irradiation

The application of 2° TD for the delivery of a therapeutic vector was analyzed in vivo by testing GFP-*FANCC* expressing LV in conjunction with carrier cells followed by in vivo selection to expand the genetically corrected HSPCs. Conceptually, the chemotactic migration towards BM and the ability to shield the vector particles from neutralizing antibodies suggests that hematopoietic progenitors can be a suitable model to test in vivo delivery of LV particles [19]. We used hematopoietic progenitors as carriers derived from Tomato-positive transgenic B57BL/6 J mice (Tomato HSPCs) for the delivery of LV-GFP-FANCC vector to the recipient cells (CD45.1 HSPCs) [19]. The 2° TD events were measured by GFP expression (FACS) and colony-forming assay to confirm the intercellular transfer of LV between the HSPCs. Consistent with earlier results, MMC exposure of the 2° target improved post-transduction transgene expression suggesting the protein response to induced DNA damage (Fig. 3a) [23]. Vector transfer between carrier and recipient HSPCs was further analyzed by colony-forming assay which allowed us to distinguish colony-forming units from both 1° (Tomato + GFP) versus 2° TD (GFP) events (Fig. 3b).

Finally, we determined in situ transduction rates in vivo in *Fancc* KO (*Fancc* ^–/–^) recipients (CD45.2) following low-dose ionizing radiation (200 cGy) conditioning to induce SDF-1α release and carrier cell homing to the BM. We used lineage depleted (Lin–) CD45.1 progenitors as carrier cells, irradiated at 1000 cGy to avoid post-delivery persistence, and exposed these irradiated carrier cells to LV-GFP-FANCC vector at MOI 20 for 6 h (Fig. 3c). To determine bone marrow homing of vector-bound, irradiated HSPCs, we measured CD45.1 chimerism (carrier) versus the endogenous recipient cells (CD45.2 *Fancc* ^*–/–*^) in the peripheral blood (Fig. 3d). Predictably, recipient cells represented a dominant fraction in transplanted animals and indicate the intended loss of irradiated carrier cells as well as vector delivery to endogenous targets in the bone marrow. To further boost the percentage of phenotypically corrected *Fancc* ^*–/–*^ HSPCs, we used serial low doses of post-transplant ionizing radiation [24]. Remarkably, the 2° TD *Fancc* ^*–/–*^ KO animals showed a consistently expanding population of GFP-FANCC expressing cells (Fig. 2e), illustrating the in vivo selection and expansion of FA HSPCs via 2° TD.

## Discussion

The same ex vivo manipulation of HSPCs successfully utilized in several hematopoietic gene therapy trials appears to impair the proliferation and repopulation potential of FA HSPCs [25, 26]. An alternative direct inoculation of vector into the bone marrow for transduction of host cells, on the other hand, risks complement neutralization and the possibility of eliciting an immune response [27]. By contrast, carrier cell surface bound vector particles appear to escape neutralization by serum complement [18], and the in situ gene delivery into the bone marrow stem cell compartment using carrier cells has not been previously attempted. Several underlying principles of systemic delivery and distant target cell transduction have already been validated for oncolytic virotherapy, where antigen-specific (for homing) T cells delivered oncolytic measles virus particles to control metastatic growth [28, 29]. Combining these principles with LV hitchhiking, i.e., cellular vector capture by carrier cells, ex vivo protection from elimination by the host innate immune surveillance, chemotaxis to the BM microenvironment, and in situ delivery to BM stem cells may confer functional correction in FA [30]. We systematically simulated the critical aspects of in situ gene delivery, including secondary transduction and clearance of carrier cells. Considerable evidence supports the involvement of CXCR4-SDF-1α as the critical axis for homing to the bone marrow compartment [31–33]. In proof of principle studies, we used low-dose radiation to elicit SDF-1α-induced homing of carrier HSPCs to the bone marrow to mediate in situ delivery of the therapeutic LV-GFP-FANCC vector to endogenous *Fancc* ^–/–^ HSPCs. Our model relies on congenic HSPCs as carriers for vector delivery to recipient HSPCs. For clinical translation, non-radiation based strategies, including inducible suicide genes (HSVtk, iCaspase-9), would be preferable to effectively eliminate the carrier cell population in vivo [34]. Finally, the in vivo selection of a small number of transduced cells is a critical component of our approach. Clonal expansion of spontaneously corrected HSPCs under naturally occurring positive selection suggests that a minimal number of corrected cells can lead to clinical reversal of cytopenias in FA patients [19–21]. Hence, we propose that a small population of functionally corrected FA HSPCs via 2° TD can restore hematopoiesis in the FA patients using optimal in vivo selection methods [35]. In aggregate, the described approach of in situ cellular delivery and phenotypic correction of HSPCs may be suitable to incorporate while designing gene therapy protocols for FA.

## Additional file

Additional file 1: Materials and methods. (DOCX 16 kb)

## Abbreviations

BM: Bone marrow
FA: Fanconi anemia
GFP: Green fluorescent protein
HSC: Hematopoietic stem cell
HSPC: Hematopoietic stem and progenitor cell
LV: Lentiviral vector
MMC: Mitomycin-C
SDF-1α: Stromal-derived factor-1α
TD: Transduction
