# Supplementary material for: Phenotypic correction of Fanconi anemia cells in the murine bone marrow after carrier cell mediated delivery of lentiviral vector
Source: Stem Cell Res Ther. 2016 Nov 19;7:170. doi: 10.1186/s13287-016-0431-z (PMC5116221; doi:10.1186/s13287-016-0431-z)
Supplement: Additional file 1: — Materials and methods. (DOCX 16 kb) [file 13287_2016_431_MOESM1_ESM.docx]

**Additional file 1: Materials & Methods**

**Plasmids and Vector production**

The 3^rd^ generation HIV-1 based transfer plasmid pWPXL was used to incorporate the 2 kb GFP FANCC ORF fragment. In the 4-plasmid vector system used in this study, pLVCG was used with the helper plasmids pLP1 (coding HIV-1 Gag-Pol), pLP2 (coding HIV-1 Rev) and pLP/VSVG (encoding Vesicular Stomatitis Viral envelope glycoprotein) (GIBCO-Invitrogen) to produce the vector particles. The integrating viral vector stocks were produced by using the helper constructs pLP1, pLP2 and pLP/VSVG. Calcium phosphate transfection was performed in the presence of DMEM (Gibco), 10% FBS (Gibco), 1% Penicillin/ Streptomycin (Pen/Strep, Gibco). Vector supernatant was harvested 36, 48, and 72 hours later, filtered through a 0.45 μm filter, pooled and ultra-concentrated over 30 hours at 7300 RCF, and the pellet was resuspended in Iscove’s media (Gibco) and stored at -86°C until use. Limiting dilution titers were determined by FACS and calculated using 293T cells, as previously described [17]

**Cell culture**

HEK 293T human kidney fibroblasts and PD331 cells were grown in DMEM supplemented with 10% FBS and 1% Pen/Strep. L1210 cells were grown in RPMI supplemented with 10% FBS and 1% Pen/Strep. HPCs were cultured in IMDM medium containing 10% fetal bovine serum, 10% horse serum, and 1% Penicillin /Streptomycin, as previously described. In co-culture experiments, Carrier cells incubated with GFP expressing lentiviral vector LVCG at MOI 1 for 3 hours were washed twice and then incubated overnight with recipient cells at a 1:1 ratio. GFP expression from wild type recipient cells indicates vector transfer from carrier cells and stable proviral integration in recipient cells. With a preclinical setting in mind, we reasoned that the elimination of carrier cells after vector delivery would avoid persistence and reduce the emergence of a potential immune response in this *in situ* model of gene delivery. To confirm that permanently arrested cells similarly transmit LV particles to other cells, we irradiated carrier cells prior to incubation with LV particles.

**Animal husbandry**

C57BL/6, Fancc ^-/-^ and mT/mG mice were group-housed and allowed *ad libitum* access to standard chow pellets (Purina Laboratory Rodent Diet 5001; Ralston Purina Co., MO, USA). Whole bone marrow cells were collected by flushing femurs and tibias from 8- to 12-week-old (CD45.1/2) with Iscove's modified Dulbecco's media. Samples were depleted of red cells by hemolysis and lineage-depleted using an Easy Sep Mouse Hematopoietic Progenitor Cell Enrichment kit according to manufacturer's instructions (StemCell Technologies Inc., Vancouver, Canada). Following LV transduction at MOI 10-20 in the presence of 8μg/ml protamine sulfate, cells were incubated overnight and washed twice in PBS, resuspended in 200 μL Hank's balanced salt solution, and injected intravenously into myeloablated (250 cGy) recipients. Following transplantation, retro-orbital eye bleeds were performed at intervals, and white blood cells were analyzed for transgene expression by flow-cytometry. HSPCs derived from the mT/mG mice were used as carrier cells in the *in vitro* experiments. All animal studies were approved by the OHSU institutional animal care and use committee.

**Flow-cytometry**

GFP, Tomato and DsRed expression was analyzed with a FACS-Calibur and Canto-II instrument (BD Biosciences) and processed using Flow Jo software (Tree Star, Ashland, OR). At least ten thousand events were collected for any given experiment. Mean fluorescence intensities (MFI) were also analyzed using Flow Jo software. The software determines the test result by comparing the MFI of individual cells against total population of GFP positive cells. For stringent clonal selection, florescent reporter protein positive cells were sorted by InFlux Cell Sorter (BD Biosciences). Sorted cells were washed twice in DMEM and propagated in 10 cm plates at 100 cells per plate.

**PCR**

Polymerase Chain Reaction (PCR) was used to analyze the LV transduced cellular DNA. 25uL reactions were set up by adding DNA template followed by 1X Taq PCR buffer, 200uM dNTPs, 0.2uM primers and 1.25 units of Taq polymerase (Invitrogen). Primer sequences used in the PCR reactions are PPT F (5’-acaaggcagctgtagatcttagccac-3’) and GFP R (5’-ttcaccggggtgtgcccatcctg-3’). Thermocycler conditions: denaturation at 95^o^C for 5 minutes, followed by 35 cycles denaturation (95^o^C – 1 minute), annealing (65^o^C – 1 minute) and elongation (72^o^C – 5 minutes). Terminal elongation at 72^o^C for 10 minutes was performed followed by hold temperature at 12^o^ C. Reactions were analyzed by agarose gel electrophoresis.

**Statistics**

Statistical significance was determined by performing a paired 2-tailed Student’s t-test. Individual figure panels indicate whether equal or unequal variance was assumed. P-values of less than 0.05 were considered statistically significant.
